# Association Between Serum 25-Hydroxyvitamin D Levels and Neuropathic Pain Features in Adults Presenting with Musculoskeletal Pain: A Cross-Sectional Study

**DOI:** 10.3390/metabo16090667

**Published:** 2026-09-10

**Authors:** Candost Ege Satilmis, Bulent Akyuz, Serpil Demir

**Affiliations:** Department of Physical Medicine and Rehabilitation, Akdeniz University Hospital, Antalya 07058, Türkiye; drbulentakyuz@outlook.com (B.A.); serpiltuna@akdeniz.edu.tr (S.D.)

**Keywords:** vitamin D, neuropathic pain, DN4, LANSS, painDETECT, short-form McGill pain questionnaire

## Abstract

Background/Objectives: The association between vitamin D status and neuropathic pain in musculoskeletal disorders remains uncertain. This study examined the relationship between serum 25-hydroxyvitamin D [25(OH)D] levels and neuropathic pain features in adults with musculoskeletal pain. Methods: This single-center analytical cross-sectional study included 300 adults aged 18–79 years who presented between June and December 2024. Participants were classified as vitamin D deficient (<20 ng/mL; *n* = 105), insufficient (20–<30 ng/mL; *n* = 117), or sufficient (≥30 ng/mL; *n* = 78). Neuropathic pain was assessed using DN4, LANSS, painDETECT, and the Short-Form McGill Pain Questionnaire. Multivariable logistic regression adjusted for age, sex, body mass index, diabetes mellitus, inflammatory rheumatic disease, cervical/lumbar discopathy, and fibromyalgia. Results: Neuropathic pain scores increased as 25(OH)D levels decreased. DN4 positivity occurred in 53.3%, 43.6%, and 25.6% of the deficient, insufficient, and sufficient groups, respectively (*p* < 0.001). Compared with sufficiency, vitamin D deficiency and insufficiency were associated with DN4 positivity (adjusted odds ratio [aOR] 3.53, 95% confidence interval [CI] 1.76–7.08; and aOR 2.38, 95% CI 1.22–4.64, respectively). Each 10 ng/mL decrease in 25(OH)D was associated with 46% higher odds of DN4 positivity (aOR 1.46, 95% CI 1.14–1.86). Similar associations were observed for LANSS and painDETECT ≥13 and persisted in sensitivity analyses. Conclusions: Lower 25(OH)D levels were associated with higher neuropathic pain scores and more frequent positive screens. The cross-sectional design, modest effect sizes, and unmeasured supplementation and calcium–parathyroid hormone variables preclude conclusions regarding causality, diagnostic utility, or treatment efficacy.

## 1. Introduction

Neuropathic pain, defined as pain arising from a lesion or disease affecting the somatosensory nervous system, occurs across disorders including radiculopathy, diabetes mellitus, and chronic musculoskeletal conditions [1,2]. It is common and is associated with substantial functional impairment, reduced quality of life, and healthcare use [3,4]. Identifying neuropathic features may therefore complement the assessment of inflammatory and nociceptive pain mechanisms.

Serum 25-hydroxyvitamin D [25(OH)D], the predominant circulating metabolite of vitamin D, is the standard biomarker used to evaluate vitamin D status. Although serum concentrations below 20 ng/mL, between 20 and <30 ng/mL, and ≥30 ng/mL are commonly used to categorize vitamin D deficiency, insufficiency, and sufficiency, respectively, optimal thresholds remain debated, and routine screening of asymptomatic adults is not universally recommended; interpretation of vitamin D status should therefore take the clinical context and individual risk factors into account [5,6]. This distinction is important in pain research because a low 25(OH)D level may represent both a biologically meaningful exposure and a complex clinical marker reflecting sun exposure, physical activity, diet, obesity, comorbidity, and medication use.

The roles of vitamin D beyond its classical functions in calcium-phosphate and bone metabolism are receiving increasing attention in relation to the nervous system and pain biology. Vitamin D receptor-mediated signaling pathways have been proposed to be involved in neuroimmune regulation, neuronal differentiation, oxidative stress responses, neurotrophic support, and neuromuscular function [7,8]. However, this biological plausibility does not imply direct causality at the clinical level. The current clinical literature is markedly heterogeneous with respect to sample characteristics, underlying pain etiology, vitamin D thresholds, definitions of neuropathic pain, and control of confounding factors.

Most clinical studies in this field have focused on diabetic neuropathy or selected disease-specific cohorts and have often relied on a single screening instrument. Evidence from heterogeneous adults presenting with musculoskeletal pain and assessed using several complementary pain instruments is more limited. We therefore examined whether lower serum 25(OH)D levels were associated with higher neuropathic pain scores and more frequent positive screening results using DN4, LANSS, painDETECT, and the Short-Form McGill Pain Questionnaire. We hypothesized that these associations would persist after adjustment for prespecified clinical covariates.

## 2. Materials and Methods

This study was conducted using a single-center analytical cross-sectional design, with comparison groups defined according to serum 25(OH)D categories. A total of 300 consecutive eligible patients aged 18–79 years who presented with musculoskeletal pain to the Physical Medicine and Rehabilitation outpatient clinics of Akdeniz University Faculty of Medicine between June and December 2024 were included. The recruitment period encompassed summer, autumn, and early winter. Assessments were performed using a structured clinical form developed for the study and pain scales administered face-to-face. The pain questionnaires were completed before the serum 25(OH)D result was reviewed by the clinician administering the instruments.

The patients’ sociodemographic characteristics, medical histories, current medication use, comorbidities, and relevant laboratory parameters were recorded. Patients with a history of vitamin B12 deficiency, malignancy, entrapment neuropathy, spinal cord injury, chronic alcoholism, spinal stenosis, uncontrolled diabetes mellitus, active chemotherapy, or active radiotherapy were excluded. For the purposes of this study, uncontrolled diabetes mellitus was operationally defined as an HbA1c level ≥ 9.0%. These criteria were used to reduce alternative causes of neuropathic pain and allow a clearer assessment of the association between serum 25(OH)D and neuropathic pain features.

Serum 25(OH)D was the main exposure variable, and participants were stratified into three categories: deficient (<20 ng/mL), insufficient (20–<30 ng/mL), and sufficient (≥30 ng/mL). Participants with a serum 25(OH)D level of exactly 30.0 ng/mL were included in the sufficient group. Baseline variables included age, body mass index (BMI), sex, diabetes mellitus, cervical/lumbar discopathy, fibromyalgia, inflammatory rheumatic disease, antirheumatic treatment, antiresorptive drug use, and cardiovascular medications. Alkaline phosphatase, calcium, C-reactive protein, and erythrocyte sedimentation rate values were obtained from laboratory results from the same clinical assessment period. Vitamin D and calcium supplement use could not be reliably retrieved from the available records and was therefore not analyzed. Parathyroid hormone and albumin values were not systematically available across the cohort; corrected calcium could not be calculated, and a heterogeneous subset analysis was not performed.

Neuropathic pain status was primarily categorized using the DN4 tool, adopting the standard threshold of ≥4 points to denote a positive screening result [9]. As secondary clinical endpoints, continuous tracking and distinct cut-offs were applied to the remaining metrics: a score of 12 or higher established positivity on the LANSS instrument [10], whereas the painDETECT questionnaire utilized tiered boundaries, where values between 13 and 18 suggested a possible neuropathic component and scores ≥ 19 strongly indicated its presence [11]. The total Short-Form McGill Pain Questionnaire score was used for the multidimensional assessment of pain severity [12]. On all scales, higher scores were considered consistent with more pronounced pain or neuropathic pain features.

The study protocol was approved by the local ethics committee of Akdeniz University Hospital (Approval No. TBAEK-262; date: 25 April 2024). Written informed consent was obtained from all participants who agreed to take part in the study.

Continuous variables were summarized as medians and interquartile ranges, and categorical variables as counts and percentages. The Kruskal–Wallis test was used for continuous variables and Pearson’s chi-square test for categorical variables in comparisons across serum 25(OH)D categories. Effect size was reported as epsilon-squared (ε^2^) for continuous variables and Cramer’s V for categorical variables.

Multivariable logistic regression models were constructed for the primary outcome of a positive DN4 screen for neuropathic pain. In separate models, serum 25(OH)D was evaluated both as a categorical variable, with the sufficient vitamin D group (≥30 ng/mL) as the reference, and as a continuous variable, per 10 ng/mL lower serum 25(OH)D level. The models were adjusted for BMI, age, sex, diabetes mellitus, inflammatory rheumatic disease, lumbar/cervical discopathy, and fibromyalgia. Age, sex, and BMI were included as core demographic and anthropometric covariates, whereas diabetes mellitus, inflammatory rheumatic disease, cervical/lumbar discopathy, and fibromyalgia were included as clinically relevant potential confounders because of their plausible associations with neuropathic pain features and/or serum 25(OH)D levels. Covariate selection was based on clinical relevance rather than univariable statistical significance. The same adjustment set was applied in secondary models for LANSS positivity, painDETECT positivity indicating a possible/highly probable component, painDETECT positivity indicating a highly probable component, positivity on any highly probable screen, and concurrent positivity across all three highly probable screening tools.

The Benjamini–Hochberg method was used to adjust the false discovery rate (FDR) when interpreting multiple comparisons. Sensitivity analyses included a binary definition of vitamin D deficiency (<20 ng/mL vs. ≥20 ng/mL), a complete-case model with additional adjustment for CRP, additional models incorporating antiresorptive drug use, a model excluding high 25(OH)D outliers according to Tukey’s rule, and researcher-prespecified alternative model specifications. In addition, potential effect modification by sex was evaluated by including a serum 25(OH)D × sex interaction term in the primary DN4 model. For the assessment of potential selection bias in the CRP complete-case analysis, participants with and without available CRP data were compared using the Mann–Whitney U test for continuous variables and Fisher’s exact test for categorical variables. No a priori sample size calculation was performed. The sample size was determined by inclusion of all consecutive eligible patients during the study period. The primary categorical model included 127 DN4-positive events for nine predictor parameters (approximately 14 events per predictor parameter); secondary models with fewer events were considered exploratory. All primary statistical analyses were performed using IBM SPSS Statistics for Windows, Version 23.0 (IBM Corp., Armonk, NY, USA). Effect size estimates were calculated and Benjamini–Hochberg false discovery rate corrections were applied based on the final SPSS-derived statistical results. Multivariable logistic regression results were reported as adjusted odds ratios (aORs) with 95% confidence intervals (CIs). All tests were two-sided, and *p* < 0.05 was considered the threshold for statistical significance.

## 3. Results

A total of 300 patients were included in the study. According to serum 25(OH)D categories, 105 patients were in the vitamin D deficiency group, 117 in the vitamin D insufficiency group, and 78 in the sufficient vitamin D group. In the overall cohort, the median age was 51.0 years (38.0–60.0), and 24.0% of participants were male. Participants with sufficient vitamin D levels were older than those in the deficiency and insufficiency groups (*p* < 0.001; ε^2^ = 0.09). BMI, calcium, CRP, and ESR did not differ significantly among the groups. Alkaline phosphatase levels differed significantly among the groups, but the effect size was small (*p* = 0.005; ε^2^ = 0.03).

Among categorical variables, the proportion of male participants was highest in the vitamin D deficiency group and lowest in the sufficient vitamin D group (32.4%, 23.1%, and 14.1%, respectively; *p* = 0.016; Cramer’s V = 0.17). Diabetes mellitus, lumbar/cervical discopathy, fibromyalgia, and treatment for rheumatic disease did not differ significantly among the groups. Use of antiresorptive medication was more common in the sufficient vitamin D group (*p* < 0.001; Cramer’s V = 0.25). The distribution of comorbidity categories differed across serum 25(OH)D groups (*p* = 0.006; Cramer’s V = 0.16) (Table 1).

**Figure 1:**
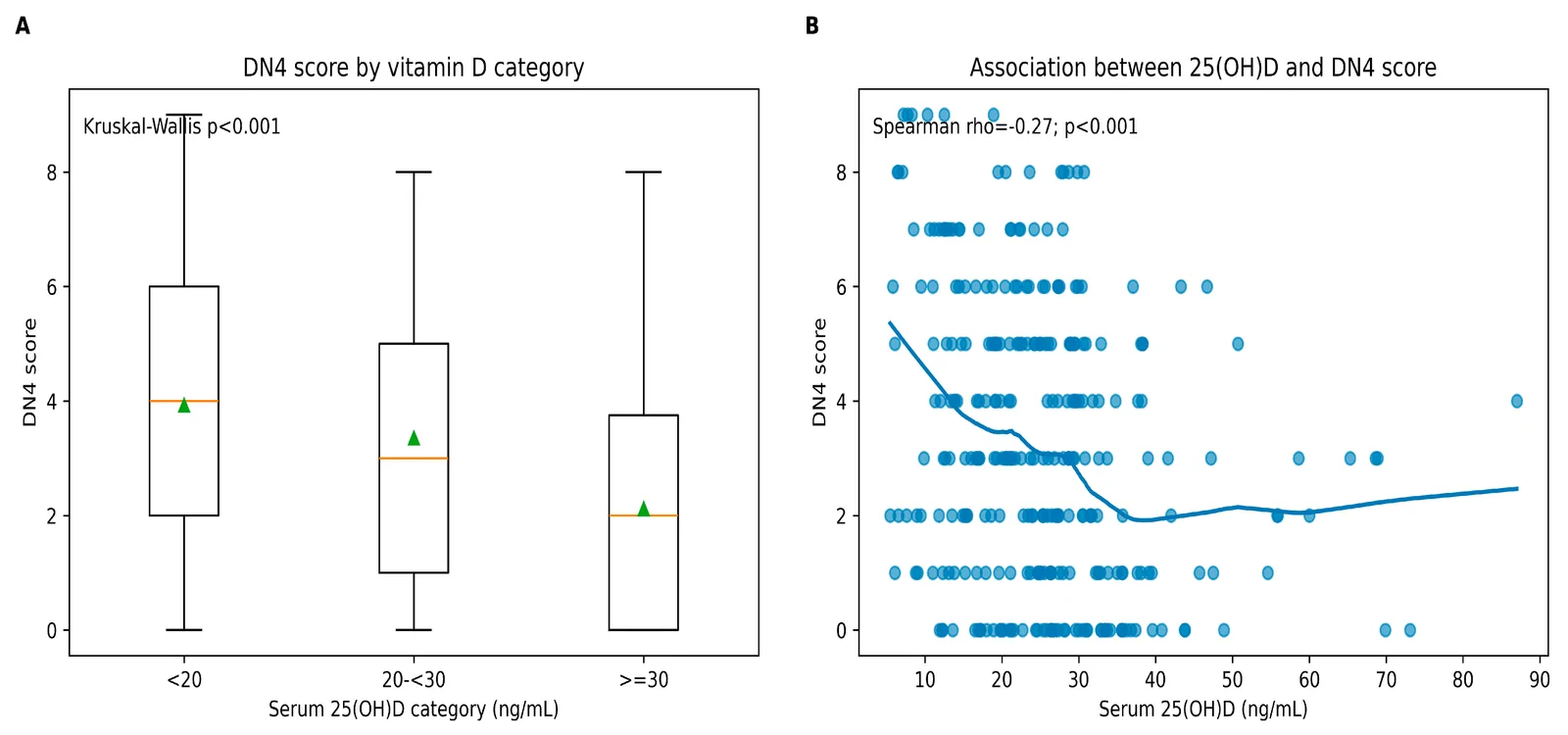
Distribution and continuous association of serum 25(OH)D with DN4 neuropathic pain score. Legend: Panel (**A**) shows DN4 score distributions across serum 25(OH)D categories. Boxes represent the interquartile range, horizontal lines represent medians, whiskers show the conventional 1.5 × IQR range, and triangle markers indicate means. Panel (**B**) shows individual observations and a locally weighted fitted curve for the association between continuous serum 25(OH)D and DN4 score. Spearman rho = −0.27; *p* < 0.001. DN4 ≥ 4 defines a positive neuropathic pain screen. Abbreviations: 25(OH)D, 25-hydroxyvitamin D; DN4, Douleur Neuropathique 4; IQR, interquartile range.

Neuropathic pain scores showed a consistent gradient across serum 25(OH)D categories. The median DN4 score was 4.0 (2.0–6.0) in the deficiency group, 3.0 (1.0–5.0) in the insufficiency group, and 2.0 (0.0–3.8) in the sufficient vitamin D group (*p* < 0.001; FDR q < 0.001). Similarly, total LANSS, total painDETECT, and total Short-Form McGill Pain Questionnaire scores were higher in the deficiency/insufficiency groups than in the sufficient vitamin D group (all *p* < 0.001; FDR q ≤ 0.001). A weak-to-moderate negative correlation was observed between serum 25(OH)D levels and the DN4 score (Spearman rho = −0.27; *p* < 0.001) (Figure 1).

Screen positivity was also more frequent at lower 25(OH)D levels. DN4 positivity was observed in 56/105 (53.3%) participants in the deficiency group, 51/117 (43.6%) in the insufficiency group, and 20/78 (25.6%) in the sufficient vitamin D group (*p* < 0.001; FDR q = 0.003). LANSS positivity rates were 27.6%, 20.5%, and 9.0%, respectively (*p* = 0.008; FDR q = 0.014). painDETECT ≥ 13 positivity rates were 42.9%, 37.6%, and 21.8% in the deficiency, insufficiency, and sufficient groups, respectively (*p* = 0.010; FDR q = 0.014). In contrast, positivity for a highly probable neuropathic pain component according to the painDETECT ≥ 19 threshold did not reach statistical significance across groups (*p* = 0.143) (Table 2).

In the categorical primary model, vitamin D deficiency and insufficiency were associated with DN4 positivity relative to sufficiency (aOR 3.53, 95% CI 1.76–7.08, *p* < 0.001; and aOR 2.38, 95% CI 1.22–4.64, *p* = 0.011, respectively) (Table 3A). In the separate continuous model, each 10-ng/mL lower 25(OH)D level was associated with higher odds of DN4 positivity (aOR 1.46, 95% CI 1.14–1.86, *p* = 0.002) (Table 3B). Inflammatory rheumatic disease was inversely associated with DN4 positivity in the categorical model (aOR 0.47, 95% CI 0.23–0.95, *p* = 0.035), but the estimate was attenuated in the continuous model (aOR 0.52, 95% CI 0.26–1.05, *p* = 0.067).

**Figure 2:**
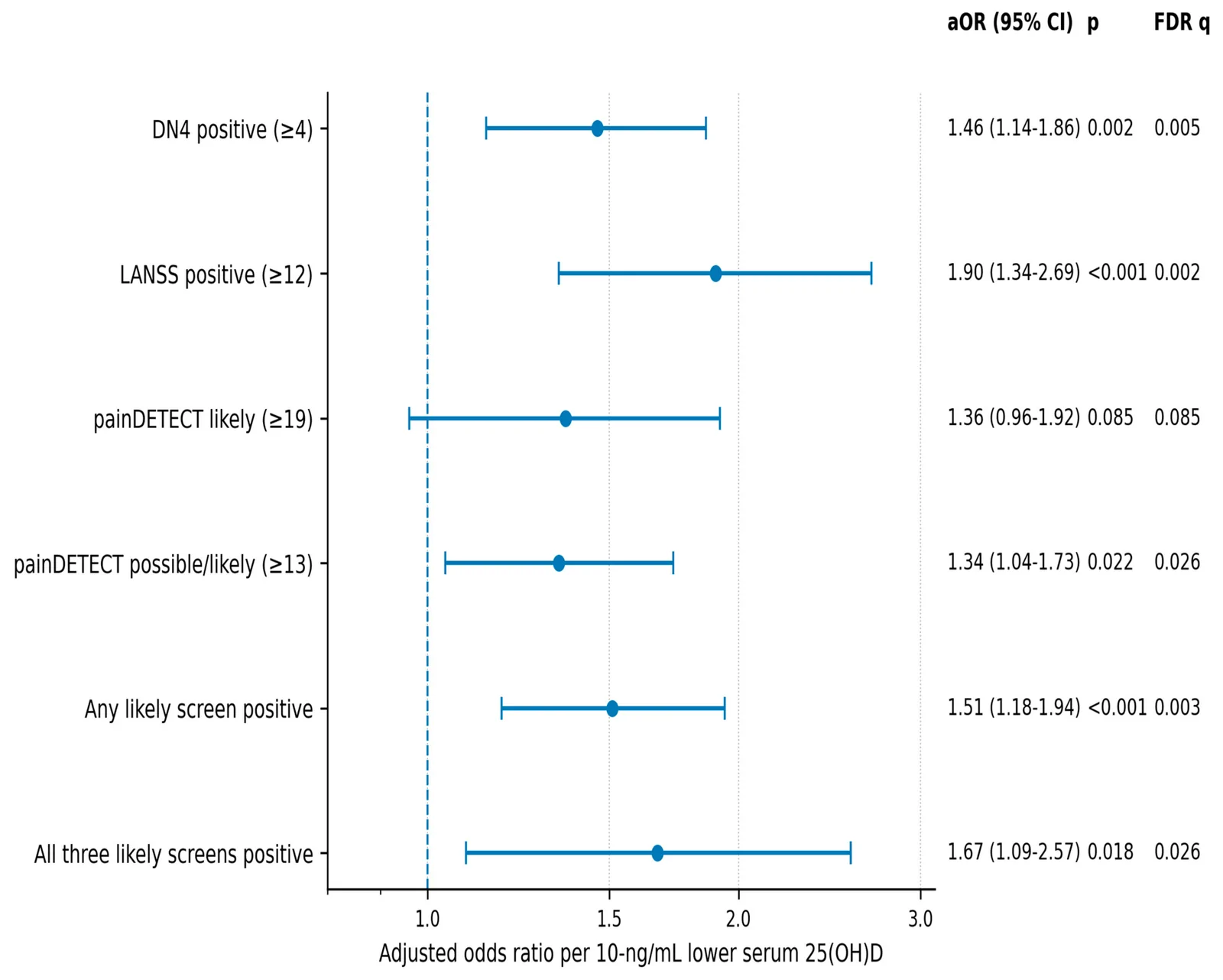
Adjusted odds ratios for neuropathic pain screening outcomes per 10-ng/mL lower serum 25(OH)D. Legend: Points represent adjusted odds ratios and horizontal lines represent 95% confidence intervals. The dashed vertical line indicates no association (aOR = 1). Models were adjusted for age, sex, BMI, diabetes mellitus, inflammatory rheumatic disease, lumbar/cervical discopathy, and fibromyalgia. FDR q values were calculated across the adjusted secondary model family using the Benjamini–Hochberg method. Abbreviations: 25(OH)D, 25-hydroxyvitamin D; aOR, adjusted odds ratio; BMI, body mass index; CI, confidence interval; FDR, false discovery rate; LANSS, Leeds Assessment of Neuropathic Symptoms and Signs.

Secondary adjusted models supported a generally consistent association between lower serum 25(OH)D levels and different screening outcomes. Each 10 ng/mL lower 25(OH)D level was associated with LANSS positivity (aOR 1.90; 95% CI 1.34–2.69; *p* < 0.001; FDR q = 0.002), painDETECT ≥ 13 positivity (aOR 1.34; 95% CI 1.04–1.73; *p* = 0.022; FDR q = 0.026), positivity on any highly probable screen (aOR 1.51; 95% CI 1.18–1.94; *p* < 0.001; FDR q = 0.003), and concurrent positivity on all three highly probable screens (aOR 1.67; 95% CI 1.09–2.57; *p* = 0.018; FDR q = 0.026). The association for the painDETECT ≥ 19 threshold did not reach statistical significance (aOR 1.36; 95% CI 0.96–1.92; *p* = 0.085; FDR q = 0.085) (Figure 2).

Sensitivity analyses were consistent with the main findings in direction and magnitude. When vitamin D deficiency was defined dichotomously as <20 ng/mL versus ≥20 ng/mL, it was associated with DN4 positivity (aOR 1.95; 95% CI 1.18–3.25; *p* = 0.010). In the complete-case model with additional adjustment for CRP, the aOR per 10 ng/mL lower 25(OH)D level was 1.47 (95% CI 1.15–1.89; *p* = 0.002). Additional adjustment for antiresorptive drug use did not materially alter the association. Compared with the vitamin D-sufficient group, the aORs for DN4 positivity were 3.46 (95% CI 1.72–6.96; *p* < 0.001) for vitamin D deficiency and 2.33 (95% CI 1.19–4.57; *p* = 0.014) for vitamin D insufficiency. In the corresponding continuous model, each 10-ng/mL lower serum 25(OH)D level remained associated with higher odds of DN4 positivity (aOR 1.44; 95% CI 1.13–1.85; *p* = 0.003) (Table 4 and Supplementary Table S1). There was no statistical evidence of effect modification by sex in the association between serum 25(OH)D levels and DN4 positivity (25(OH)D × sex interaction, *p* = 0.376). CRP was available for 286 participants and missing for 14. No statistically detectable differences were observed between participants with and without available CRP data in age, sex, BMI, serum 25(OH)D level, DN4 score, or DN4 positivity, although the small number of participants with missing CRP data limited the precision of this comparison (Supplementary Table S2). The association was also retained after exclusion of high 25(OH)D outliers (aOR 1.56; 95% CI 1.17–2.08; *p* = 0.003). The compatibility models used a different covariate set and expressed effects per 1-ng/mL higher 25(OH)D, whereas the primary models expressed effects per 10-ng/mL lower 25(OH)D. The estimates therefore have opposite directions of scaling and should not be compared numerically without accounting for these differences. In the compatibility models, each 1 ng/mL higher 25(OH)D level was associated with lower odds of DN4 positivity (aOR 0.96; 95% CI 0.94–0.99; *p* = 0.002) and lower odds of LANSS positivity (aOR 0.94; 95% CI 0.91–0.98; *p* = 0.001) (Table 4).

## 4. Discussion

In this study, low serum 25(OH)D levels were consistently associated with more pronounced neuropathic pain features in adults presenting with musculoskeletal pain. Vitamin D insufficiency and deficiency were associated with higher adjusted odds of DN4 positivity than sufficient vitamin D levels; this association persisted when serum 25(OH)D was modeled as a continuous variable. Similar directional gradients were also observed in the DN4, LANSS, painDETECT, and Short-Form McGill scores. The similar results obtained in sensitivity analyses involving CRP adjustment, exclusion of outliers, and alternative modeling approaches suggest that the observed association was not dependent on a single statistical definition.

An important aspect of the study is that neuropathic pain was assessed not with a single scale but with instruments that have different sensitivity and specificity profiles. DN4 positivity was more frequent than LANSS positivity in the sample, whereas the association did not reach statistical significance at a stricter threshold such as painDETECT ≥ 19. This pattern may be expected because the screening tools capture the same clinical construct with incomplete overlap. Ríos-León et al. reported that DN4 had the highest sensitivity, whereas S-LANSS and painDETECT had higher specificity in acute whiplash-associated disorders, providing a useful framework for interpreting the broader positivity pattern observed with DN4 and the more selective positivity patterns observed with LANSS and painDETECT in our study [13]. Therefore, while our results support an association between low 25(OH)D levels and neuropathic pain features, they also emphasize that neither serum 25(OH)D nor a single questionnaire score should be regarded as a diagnostic test for neuropathic pain.

The statistical associations were accompanied by modest effect sizes. Across pain scores, epsilon-squared values ranged from 0.04 to 0.08, and the Spearman correlation between 25(OH)D and DN4 was −0.27. Thus, vitamin D status accounted for only a limited component of the observed pain phenotype and is unlikely to provide adequate individual-level discrimination on its own. Statistical significance in a sample of 300 should therefore not be interpreted as evidence of a large clinical effect. Our findings are consistent with studies reporting an association between low vitamin D levels and neuropathic pain in different clinical contexts. From a rheumatological perspective, a study reporting an association between serum vitamin D levels and neuropathic pain features in patients with rheumatoid arthritis showed that the vitamin D-neuropathic pain axis warrants investigation even in the setting of inflammatory disease [14]. In the diabetic neuropathy literature, low 25(OH)D levels have been associated with the painful phenotype and small-fiber-related findings in well-phenotyped groups with painful diabetic peripheral neuropathy [15,16]. The study by Al Ali et al. involving 600 patients with type 2 diabetes also supported a possible association between vitamin D deficiency and diabetic neuropathy [17]. The review by Putz et al. emphasized that evidence has accumulated across different complication domains, including peripheral neuropathy, diabetic foot, and cardiovascular autonomic neuropathy, but that stronger study designs are needed before direct translation into clinical practice [18]. The fact that our cohort did not consist solely of individuals with diabetes provides a distinct contribution by demonstrating the association in a more heterogeneous population with musculoskeletal pain.

Nevertheless, the literature is not entirely consistent. Alkhatatbeh and Abdul-Razzak reported that neuropathic pain in patients with type 2 diabetes mellitus was associated with female sex rather than serum vitamin D levels [19]. Similarly, a recent study in patients with lipedema found no significant association of 25(OH)D or vitamin B12 levels with neuropathic pain [20]. These conflicting results suggest that the vitamin D-neuropathic pain relationship may be sensitive to disease-specific pain mechanisms, sex distribution, BMI, sun exposure, physical activity, supplement use, ethnic/geographical differences, and the screening instrument used. Although adjustment for clinical variables such as age, sex, BMI, diabetes, discopathy, and fibromyalgia is an important feature of our study, residual confounding cannot be completely excluded.

From the perspective of the hierarchy of evidence, an observational association is not equivalent to evidence of treatment benefit. The meta-analysis by Yammine et al. on vitamin D status and diabetic neuropathy reported that low vitamin D levels may be more common among individuals with diabetic neuropathy [21]. A systematic review by the same research group on the efficacy of vitamin D replacement in diabetic peripheral neuropathy emphasized that supplementation may provide additional benefit in reducing pain, but that the evidence is limited by the number and design quality of available studies [22]. A more recent meta-analysis of randomized trials also showed that vitamin D may reduce short-term pain in painful diabetic neuropathy, although the evidence should be interpreted cautiously because of small sample sizes and methodological heterogeneity [23]. Although the studies by Pinzon, Shehab, and Basit reported improvements in pain or neuropathy symptoms after vitamin D administration, their patient populations predominantly comprised individuals with diabetic neuropathy, and their results cannot be directly generalized to all populations presenting with musculoskeletal pain [24,25,26]. Therefore, our study supports an association between vitamin D status and the neuropathic pain phenotype rather than the efficacy of replacement therapy.

Potential mechanisms are likely multifaceted and cannot be established with this study design. The demonstration of the vitamin D receptor and 1-alpha-hydroxylase in neurons and glial cells of the human brain provides a structural basis for potential local biological effects of vitamin D within the nervous system [27]. Earlier experimental and translational studies suggested that vitamin D may influence neural tissue function through neurotrophic signaling, calcium homeostasis, oxidative stress, and immunomodulation [28]. The enhancement of myelination and functional recovery by cholecalciferol in models of peripheral nerve injury and the association of vitamin D deficiency with sensory hyperinnervation and hypersensitivity in skeletal muscle are experimental examples supporting this biological plausibility [29,30]. More recently, Zhang et al. showed in a model of neuropathic pain that vitamin D3 may suppress spinal GABAergic interneuron loss and mitochondria-associated ferroptosis through VDR activation, providing a new mechanistic explanation involving oxidative cellular injury and inhibitory pain modulation [31]. In addition, the association of the VDR FokI polymorphism with painful diabetic neuropathy and the association of vitamin D deficiency particularly with large-fiber lesions in older patients with type 2 diabetes suggest that individual genetic susceptibility and fiber-type phenotype should be considered in future research [32,33]. However, most mechanistic evidence is experimental or derived from diabetic neuropathy, and its applicability to the heterogeneous musculoskeletal pain population examined in the present study remains uncertain. These mechanisms should therefore be considered as providing biological plausibility rather than establishing a causal pathway underlying the observed association. The inverse association between inflammatory rheumatic disease and DN4 positivity was unexpected and should be considered exploratory. Only 46 participants had an inflammatory rheumatic disease, and the estimate differed between the categorical and continuous exposure models. Disease activity, detailed anti-inflammatory treatment exposure, and pain mechanisms within this subgroup were not characterized sufficiently to support a protective interpretation. Chance, treatment-related confounding, referral patterns, and residual differences in pain phenotype are alternative explanations.

Strengths of the study include the relatively large sample, evaluation of vitamin D status as both a categorical and continuous variable, use of multiple neuropathic pain scales, construction of models adjusted for clinically meaningful confounders, FDR correction, and a range of sensitivity analyses. Completion of questionnaires before clinicians reviewed 25(OH)D results reduced the likelihood that knowledge of the laboratory value influenced questionnaire administration. Nevertheless, the limitations should be considered. The design is cross-sectional; therefore, it cannot be determined whether low vitamin D levels contribute to neuropathic pain, whether reduced mobility and sun exposure due to pain lead to low vitamin D levels, or whether both are influenced by common determinants. The study was conducted at a single center, and because the study population consisted of patients presenting with musculoskeletal pain, the findings cannot be directly generalized to the general population or to specific neuropathy cohorts in neurological settings. No a priori sample size calculation was performed, and secondary models with fewer outcome events should therefore be interpreted as exploratory.

Furthermore, the assessment of neuropathic pain was based on clinical screening scales; definite neuropathic pain grading based on nerve conduction studies, quantitative sensory testing, skin biopsy, or a detailed neurological examination was not performed. Sun exposure, season, dietary vitamin D intake, regular vitamin D and calcium supplementation, physical activity, pain duration, analgesic use, and psychosocial variables were not quantified in detail. Recruitment from June through December encompassed several seasons, but exact month-specific sun exposure and seasonal behavior were not quantified or modeled. Parathyroid hormone and albumin were not systematically available, and corrected calcium could therefore not be calculated. Consequently, the potential contribution of calcium–parathyroid hormone homeostasis to the observed association could not be evaluated, and we cannot determine whether the association with serum 25(OH)D is independent of these metabolic pathways. In particular, the higher prevalence of antiresorptive therapy in the vitamin D-sufficient group may reflect differences in bone-health management, including potentially unmeasured vitamin D and calcium supplementation, and may therefore introduce residual confounding. Although the CRP complete-case analysis was supported by a comparison of participants with and without CRP measurements, the small missing subgroup means that selection bias cannot be excluded. These findings demonstrate an association within this cohort but do not establish clinical utility. They do not support using serum 25(OH)D as a diagnostic test for neuropathic pain, routine testing solely because neuropathic features are present, or empirical supplementation as an analgesic intervention.

Future studies should be designed as multicenter prospective cohorts that jointly evaluate baseline vitamin D status, sun exposure, supplement use, pain duration, objective neuropathy tests, and longitudinal changes in pain. Well-designed randomized controlled trials evaluating the effects of replacement therapy on pain scores, function, sleep, quality of life, and objective neuropathy parameters in subgroups with vitamin D deficiency and neuropathic pain features will clarify the clinical applicability of the observed association.

## 5. Conclusions

Lower serum 25(OH)D levels were associated with higher neuropathic pain screening scores and more frequent positive screening results in adults presenting with musculoskeletal pain. The association was consistent across several analyses but was modest in magnitude. Because of the cross-sectional design and residual confounding, these data do not establish causality, diagnostic utility, or a therapeutic benefit of vitamin D supplementation.

## Figures and Tables

**Table 1:** Baseline demographic and clinical characteristics according to serum 25-hydroxyvitamin D category.

Characteristic	Overall	Deficiency (<20)	Insufficiency (20–<30)	Sufficient (≥30)	*p* Value	Effect Size
N	300	105	117	78		
Age, years	51.0 (38.0–60.0)	45.0 (33.0–56.0)	48.0 (37.0–61.0)	57.0 (50.5–63.8)	<0.001	ε^2^ = 0.09
BMI, kg/m^2^	26.4 (23.3–29.8)	26.4 (23.3–30.5)	26.5 (23.7–30.5)	26.2 (23.1–28.5)	0.213	ε^2^ = 0.00
25(OH)D, ng/mL	24.5 (17.0–30.4)	14.2 (11.8–17.2)	25.5 (22.8–27.4)	35.7 (32.5–43.0)	<0.001	ε^2^ = 0.88
Alkaline phosphatase, U/L	70.0 (59.0–88.8)	75.0 (65.0–95.0)	67.0 (58.0–86.0)	69.0 (56.0–82.8)	0.005	ε^2^ = 0.03
Calcium, mg/dL	9.66 (9.35–9.91)	9.56 (9.33–9.90)	9.66 (9.31–9.91)	9.74 (9.48–9.94)	0.238	ε^2^ = 0.00
CRP	1.7 (0.8–3.9)	2.2 (0.8–4.6)	1.7 (0.7–4.1)	1.5 (0.8–3.1)	0.519	ε^2^ = 0.00
ESR	11.0 (9.0–16.0)	11.0 (9.0–16.0)	11.0 (8.2–16.0)	12.0 (10.0–16.0)	0.375	ε^2^ = 0.00
Male sex	72/300 (24.0)	34/105 (32.4)	27/117 (23.1)	11/78 (14.1)	0.016	Cramer’s V = 0.17
Diabetes mellitus	25/300 (8.3)	7/105 (6.7)	10/117 (8.5)	8/78 (10.3)	0.682	Cramer’s V = 0.05
Any inflammatory rheumatic disease	46/300 (15.3)	15/105 (14.3)	24/117 (20.5)	7/78 (9.0)	0.085	Cramer’s V = 0.13
Lumbar/cervical discopathy	54/300 (18.0)	18/105 (17.1)	24/117 (20.5)	12/78 (15.4)	0.633	Cramer’s V = 0.06
Fibromyalgia	9/300 (3.0)	1/105 (1.0)	4/117 (3.4)	4/78 (5.1)	0.247	Cramer’s V = 0.10
Antihypertensive/antidiabetic/antihyperlipidemic therapy	86/300 (28.7)	25/105 (23.8)	36/117 (30.8)	25/78 (32.1)	0.386	Cramer’s V = 0.08
Antiresorptive drug use	39/300 (13.0)	6/105 (5.7)	12/117 (10.3)	21/78 (26.9)	<0.001	Cramer’s V = 0.25
Treatment for rheumatic disease	46/300 (15.3)	15/105 (14.3)	22/117 (18.8)	9/78 (11.5)	0.361	Cramer’s V = 0.08
Comorbidity category: no comorbidity	100/300 (33.3)	46/105 (43.8)	34/117 (29.1)	20/78 (25.6)	0.006	Cramer’s V = 0.16
Comorbidity category: other comorbidity	154/300 (51.3)	44/105 (41.9)	59/117 (50.4)	51/78 (65.4)		
Comorbidity category: inflammatory rheumatic disease	46/300 (15.3)	15/105 (14.3)	24/117 (20.5)	7/78 (9.0)		

Notes: Values are median (IQR) or n/N (%), unless otherwise indicated. Serum 25(OH)D categories were defined as deficiency <20 ng/mL, insufficiency 20–<30 ng/mL, and sufficient ≥30 ng/mL; two participants with exactly 30.0 ng/mL were assigned to the sufficient category. Continuous variables were compared using Kruskal–Wallis tests and categorical variables using Pearson chi-square tests. Effect sizes are epsilon-squared (ε^2^) for Kruskal–Wallis tests and Cramer’s V for categorical tests. Abbreviations: 25(OH)D, 25-hydroxyvitamin D; BMI, body mass index; CRP, C-reactive protein; ESR, erythrocyte sedimentation rate; IQR, interquartile range.

**Table 2:** Core neuropathic pain scores and screening outcomes according to serum 25(OH)D category.

Measure	Overall	Deficiency (<20)	Insufficiency (20–<30)	Sufficient (≥30)	*p* Value	Effect Size	FDR q
DN4 score	3.0 (1.0–5.0)	4.0 (2.0–6.0)	3.0 (1.0–5.0)	2.0 (0.0–3.8)	<0.001	ε^2^ = 0.07	<0.001
LANSS total score	5.0 (0.0–11.0)	6.0 (3.0–13.0)	6.0 (0.0–11.0)	2.5 (0.0–6.0)	<0.001	ε^2^ = 0.08	<0.001
painDETECT total score	9.0 (4.0–15.0)	11.0 (6.0–16.0)	11.0 (5.0–16.0)	4.0 (2.0–11.0)	<0.001	ε^2^ = 0.06	<0.001
SF-McGill total score	17.0 (8.0–26.0)	20.0 (10.0–27.0)	17.0 (8.0–27.0)	12.0 (4.0–20.0)	<0.001	ε^2^ = 0.04	0.001
DN4 positive (score ≥ 4)	127/300 (42.3)	56/105 (53.3)	51/117 (43.6)	20/78 (25.6)	<0.001	Cramer’s V = 0.22	0.003
LANSS positive (score ≥ 12)	60/300 (20.0)	29/105 (27.6)	24/117 (20.5)	7/78 (9.0)	0.008	Cramer’s V = 0.18	0.014
painDETECT possible/likely (score ≥ 13)	106/300 (35.3)	45/105 (42.9)	44/117 (37.6)	17/78 (21.8)	0.010	Cramer’s V = 0.17	0.014
painDETECT likely (score ≥ 19)	48/300 (16.0)	19/105 (18.1)	22/117 (18.8)	7/78 (9.0)	0.143	Cramer’s V = 0.11	0.143

Notes: Values are median (IQR) or n/N (%). Kruskal–Wallis tests were used for score comparisons and Pearson chi-square tests for screening classifications. FDR q values were calculated separately within the continuous score family and within the binary screening outcome family using the Benjamini–Hochberg method. Higher values indicate worse pain or greater neuropathic pain features for all listed scores. Abbreviations: 25(OH)D, 25-hydroxyvitamin D; DN4, Douleur Neuropathique 4; FDR, false discovery rate; IQR, interquartile range; LANSS, Leeds Assessment of Neuropathic Symptoms and Signs; SF-McGill, Short-Form McGill Pain Questionnaire.

**Table 3:** (**A**) Categorical multivariable model for DN4-positive neuropathic pain screening. (**B**) Continuous multivariable model for DN4-positive neuropathic pain screening.

**(A)**		
**Variable**	**aOR (95% CI)**	***p* Value**
25(OH)D ≥ 30 ng/mL	Reference	
25(OH)D < 20 vs. ≥30 ng/mL	3.53 (1.76–7.08)	<0.001
25(OH)D 20 to <30 vs. ≥30 ng/mL	2.38 (1.22–4.64)	0.011
Age, per year	1.00 (0.98–1.01)	0.634
Male sex	0.60 (0.33–1.06)	0.079
BMI, per kg/m^2^	1.02 (0.97–1.07)	0.521
Diabetes mellitus	0.73 (0.29–1.81)	0.499
Inflammatory rheumatic disease	0.47 (0.23–0.95)	0.035
Lumbar/cervical discopathy	1.25 (0.67–2.32)	0.477
Fibromyalgia	0.86 (0.19–3.79)	0.837
**(B)**		
**Variable**	**aOR (95% CI)**	***p*** ** Value**
25(OH)D, per 10-ng/mL lower	1.46 (1.14–1.86)	0.002
Age, per year	0.99 (0.98–1.01)	0.528
Male sex	0.63 (0.36–1.12)	0.114
BMI, per kg/m^2^	1.02 (0.97–1.08)	0.395
Diabetes mellitus	0.71 (0.29–1.76)	0.461
Inflammatory rheumatic disease	0.52 (0.26–1.05)	0.067
Lumbar/cervical discopathy	1.23 (0.67–2.26)	0.513
Fibromyalgia	0.76 (0.18–3.24)	0.708

The outcome was DN4 positivity (score ≥ 4). Serum 25(OH)D was entered categorically with ≥30 ng/mL as the reference. The model was adjusted for age, sex, BMI, diabetes mellitus, inflammatory rheumatic disease, lumbar/cervical discopathy, and fibromyalgia. N/events = 300/127. The outcome was DN4 positivity (score ≥ 4). Serum 25(OH)D was entered continuously per 10-ng/mL lower level. The model was adjusted for age, sex, BMI, diabetes mellitus, inflammatory rheumatic disease, lumbar/cervical discopathy, and fibromyalgia. N/events = 300/127. Abbreviations: 25(OH)D, 25-hydroxyvitamin D; aOR, adjusted odds ratio; BMI, body mass index; CI, confidence interval; DN4, Douleur Neuropathique 4.

**Table 4:** Sensitivity and compatibility analyses for the association between serum 25(OH)D and neuropathic pain screening.

Analysis	Outcome	N/Events	Effect Definition	aOR (95% CI)	*p* Value
Sensitivity	DN4 positive	300/127	25(OH)D <20 vs. ≥20 ng/mL	1.95 (1.18–3.25)	0.010
Sensitivity	DN4 positive	286/119	Additional CRP adjustment; per 10-ng/mL lower 25(OH)D	1.47 (1.15–1.89)	0.002
Sensitivity	DN4 positive	288/125	High 25(OH)D outliers excluded; per 10-ng/mL lower 25(OH)D	1.56 (1.17–2.08)	0.003
Sensitivity	DN4 positive	300/127	Additional antiresorptive adjustment; <20 vs. ≥30 ng/mL	3.46 (1.72–6.96)	<0.001
Sensitivity	DN4 positive	300/127	Additional antiresorptive adjustment; 20 to <30 vs. ≥30 ng/mL	2.33 (1.19–4.57)	0.014
Sensitivity	DN4 positive	300/127	Additional antiresorptive adjustment; per 10-ng/mL lower 25(OH)D	1.44 (1.13–1.85)	0.003
Compatibility	DN4 positive	298/127	25(OH)D per 1-ng/mL higher	0.96 (0.94–0.99)	0.002
Compatibility	LANSS positive	298/60	25(OH)D per 1-ng/mL higher	0.94 (0.91–0.98)	0.001

Notes: DN4 positive was defined as DN4 score ≥ 4; LANSS positive was defined as LANSS score ≥ 12. Sensitivity models used the primary adjustment set unless otherwise indicated. The CRP-adjusted model used complete-case data. High serum 25(OH)D outliers were identified using Tukey’s rule. Compatibility models reproduced the author-specified adjustment strategy using age, sex, BMI, ordinal comorbidity, alkaline phosphatase, and calcium; their effects are scaled per 1-ng/mL higher 25(OH)D rather than per 10-ng/mL lower 25(OH)D. Abbreviations: 25(OH)D, 25-hydroxyvitamin D; aOR, adjusted odds ratio; BMI, body mass index; CI, confidence interval; CRP, C-reactive protein; DN4, Douleur Neuropathique 4; LANSS, Leeds Assessment of Neuropathic Symptoms and Signs.

## Data Availability

The data that support the findings of this study are available on request from the corresponding author.

## Supplementary Materials

The following supporting information can be downloaded at: https://www.mdpi.com/article/10.3390/metabo16090667/s1. Table S1: Additional antiresorptive-adjusted and sex-interaction analyses; Table S2: Comparison of participants with and without CRP measurements.

## Abbreviations

The following abbreviations are used in this manuscript:

25(OH)D	25-hydroxyvitamin D
aOR	Adjusted Odds Ratio
BMI	Body Mass Index
CI	Confidence Interval
CRP	C-Reactive Protein
DN4	Douleur Neuropathique 4
ESR	Erythrocyte Sedimentation Rate
FDR	False Discovery Rate
GABA	Gamma-aminobutyric acid
HbA1c	Glycated Hemoglobin
IQR	Interquartile Range
LANSS	Leeds Assessment of Neuropathic Symptoms and Signs
S-LANSS	Self-report Leeds Assessment of Neuropathic Symptoms and Signs
SF-McGill	Short-Form McGill Pain Questionnaire
VDR	Vitamin D Receptor
