# Supplementary material for: Association Between Serum 25-Hydroxyvitamin D Levels and Neuropathic Pain Features in Adults Presenting with Musculoskeletal Pain: A Cross-Sectional Study
Source: Metabolites. 2026 Sep 10;16(9):667. doi: 10.3390/metabo16090667 (PMC13608976; doi:10.3390/metabo16090667)
Supplement: Supplementary file 1 [file metabolites-16-00667-s001.zip › metabolites-4493548-supplementary.pdf]

Supplementary Material

Association Between Serum 25-Hydroxyvitamin D Levels and Neuropathic Pain Features in Adults Presenting With Musculoskeletal Pain: A Cross-Sectional Study

Supplementary Table S1. Additional antiresorptive-adjusted and sex-interaction analyses.

Model	Term	aOR (95% CI)	p value
Categorical model + antiresorptive	25(OH)D <20 vs ≥30 ng/mL	3.46 (1.72-6.96)	<0.001
Categorical model + antiresorptive	25(OH)D 20 to <30 vs ≥30 ng/mL	2.33 (1.19-4.57)	0.014
Categorical model + antiresorptive	Antiresorptive use	0.82 (0.36-1.85)	0.626
Continuous model + antiresorptive	25(OH)D per 10-ng/mL lower	1.44 (1.13-1.85)	0.003
Continuous model + antiresorptive	Antiresorptive use	0.84 (0.37-1.90)	0.672
Sex interaction model	25(OH)D x male sex interaction	1.35 (0.69-2.64)	0.376
Sex interaction model	Women: 25(OH)D per 10-ng/mL lower	1.39 (1.07-1.80)	0.014
Sex interaction model	Men: 25(OH)D per 10-ng/mL lower	1.87 (1.01-3.49)	0.048

All models included age, sex, BMI, diabetes mellitus, inflammatory rheumatic disease, lumbar/cervical discopathy, and fibromyalgia. The first two models additionally included antiresorptive use. The interaction model included continuous 25(OH)D (per 10-ng/mL lower), male sex, and their product term. Because the interaction test was not statistically significant, sex-specific estimates are exploratory and should not be interpreted as evidence of different effects by sex. N/events=300/127.

Supplementary Table S2. Comparison of participants with and without CRP measurements.

Characteristic	CRP available (n=286)	CRP missing (n=14)	p value
Age, years	51.5 (38.0-60.0)	40.0 (34.5-54.8)	0.135
Male sex	67/286 (23.4)	5/14 (35.7)	0.336
BMI, kg/m2	26.37 (23.31-29.76)	28.11 (23.73-32.63)	0.351
25(OH)D, ng/mL	25.0 (17.03-30.58)	20.44 (16.21-23.93)	0.077
DN4 score	3.0 (1.0-5.0)	5.0 (2.0-6.8)	0.137
DN4 positive	119/286 (41.6)	8/14 (57.1)	0.278
Diabetes mellitus	23/286 (8.0)	2/14 (14.3)	0.329
Inflammatory rheumatic disease	43/286 (15.0)	3/14 (21.4)	0.458
Lumbar/cervical discopathy	52/286 (18.2)	2/14 (14.3)	1.000
Fibromyalgia	9/286 (3.1)	0/14 (0.0)	1.000
Antiresorptive drug use	38/286 (13.3)	1/14 (7.1)	1.000

Values are median (IQR) or n/N (%). Continuous variables were compared using the Mann-Whitney U test, and categorical variables using Fisher's exact test. No statistically detectable differences were observed in the listed variables, although the small CRP-missing subgroup limited precision. Abbreviations: 25(OH)D, 25-hydroxyvitamin D; BMI, body mass index; CRP, C-reactive protein; DN4, Douleur Neuropathique 4; IQR, interquartile range.

Data availability for requested metabolic variables

Vitamin D and calcium supplement use could not be reliably retrieved from the available records. Parathyroid hormone and albumin were not systematically available across the cohort; therefore, corrected calcium and calcium-parathyroid hormone axis analyses were not performed. These unavailable variables are described as limitations in the revised manuscript and were not imputed or inferred.
